# Allocation Strategies of Carbon, Nitrogen, and Phosphorus at Species and Community Levels With Recovery After Wildfire

**DOI:** 10.3389/fpls.2022.850353

**Published:** 2022-04-11

**Authors:** Zhaopeng Song, Xuemei Wang, Yanhong Liu, Yiqi Luo, Zhaolei Li

**Affiliations:** ^1^School of Ecology and Nature Conservation, Beijing Forestry University, Beijing, China; ^2^College of Urban and Environmental Sciences, and MOE Laboratory for Earth Surface Processes, Peking University, Beijing, China; ^3^Center for Ecosystem Science and Society, Northern Arizona University, Flagstaff, AZ, United States; ^4^Interdisciplinary Research Center for Agriculture Green Development in Yangtze River Basin, College of Resources and Environment, and Academy of Agricultural Sciences, Southwest University, Chongqing, China

**Keywords:** elemental allocation, leaf, fine root, recovery periods, wildfires

## Abstract

Plant stoichiometry and nutrient allocation can reflect a plant’s adaptation to environmental nutrient changes. However, the allocation strategies of carbon (C), nitrogen (N), and phosphorus (P) between leaf and fine root in response to wildfire have been poorly studied. Our primary objective was to elucidate the trade-off of elemental allocation between above- and belowground parts in response to the soil nutrient changes after a wildfire. We explored the allocation sloping exponents of C, N, and P between leaf and fine root at the species and community levels at four recovery periods (year 2, 10, 20, and 30) after moderately severe wildfire and one unburned treatment in boreal forests in Great Xing’an Mountains, northeast China. Compared with the unburned treatment, leaf C concentration decreased and fine root C increased at year 2 after recovery. The leaf N concentration at year 10 after recovery was higher than that of unburned treatment. Plant growth tended to be limited by P concentration at year 10 after recovery. Nutrient allocation between leaf and fine root differed between species and community levels, especially in the early recovery periods (i.e., 2 and 10 years). At the community level, the nutrient concentrations of the leaf changed more as compared to that of the fine root at year 2 after recovery when the fine root nutrients changed more than those of the leaf. The different C, N, and P allocation strategies advanced the understanding of plant adaptation to soil nutrient changes during the postfire ecosystem restoration.

## Introduction

As a common disturbance factor in terrestrial ecosystems, wildfires have significant consequences for forest ecosystems (Certini, 2005; Alonso-Canas and Chuvieco, 2015). Wildfires modify the physical and chemical properties of soil and accelerate soil nutrient circulations (Adler et al., 2014; Wang et al., 2015; Holden et al., 2016; Hume et al., 2016), but the effect of wildfire on the dynamics of plant nutrients remains poorly understood. During ecosystem recovery, the stoichiometric characteristics of the fine root change (Toberman et al., 2014; Yan et al., 2016) and will subsequently regulate the stoichiometries of leaves (Liu et al., 2013; Shen et al., 2016). Plants can change the allocation strategy of carbon (C), nitrogen (N), and phosphorous (P) between above- and belowground parts (Palmroth et al., 2013; Yang et al., 2015). The dynamics of nutrients are important for ecosystem recovery after a wildfire. However, our knowledge of plant nutrient allocations during recovery periods is limited; thus, understanding plant nutrient utilization strategies during the recovery period must be deepened.

Wildfires can substantially change C, N, and P concentrations in ecosystems (Holden and Treseder, 2013; Michalzik and Martin, 2013). For example, wildfires with moderate severity can reduce understory C and N pools within a short time, and then these pools will recover in several decades (Turner et al., 2008; Nave et al., 2011). An increasing number of studies have reported that soil P concentration increases after wildfires and declines as the ecosystem recovers (Hume et al., 2016; Butler et al., 2017). In general, the alterations in the amount of soil nutrients are likely to influence plant physiological processes, which further change the C, N, and P stoichiometries of plant leaf and fine root.

**FIGURE 1:**
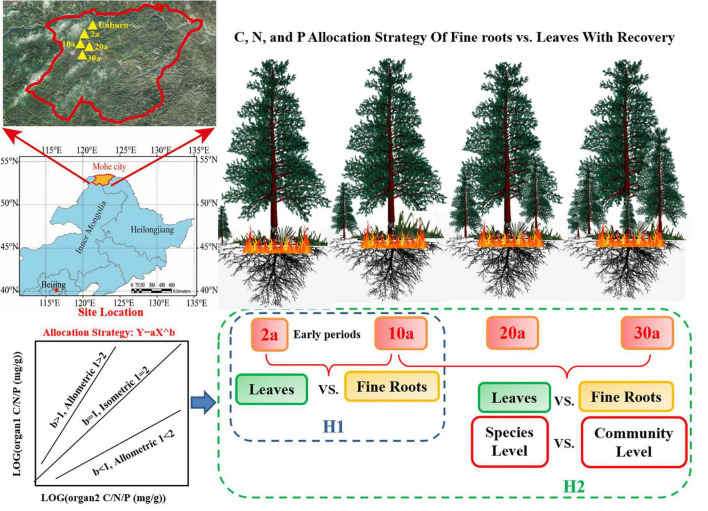
Theoretical framework for the allocation strategies of carbon (C), nitrogen (N), and phosphorus (P) with recovery after a wildfire. The plants C, N, and P stoichiometries allocation would be substantially changed in the early periods during recovery (H1), and the organ stoichiometry allocation strategies may be different in the species level and the community level (H2). 2a, 10a, 20a, and 30a are at year 2, year 10, year 20, and year 30 after recovery, respectively. UB, unburned.

Wildfire usually affects plant elemental concentrations (Hansen et al., 2016). C, N, and P are considered as the most essential elements to plant physiology (Elser et al., 2010). Few studies revealed the responses of plant physiology to ecosystem recovery. Wildfires may result in soil acidification that constrains plant uptake of P and N and change C distribution in the leaf and root (Scoffoni et al., 2011; Vernay et al., 2018). High soil nutrients can increase leaf N content and facilitate plant C synthesis in early recovery periods (approximately 10 years), which accelerates community productivity (Chen et al., 2015; Butler et al., 2017). With rapid regrowth of species after a wildfire, P availability may gradually become insufficient (Huang and Boerner, 2007; Lamont and Downes, 2011). During the recovery periods, the limitation of N and P inhibits plant growth and physiological processes, i.e., photosynthesis and respiration, which are closely related to C dynamics (LeBauer and Treseder, 2008; Chen et al., 2013). These nutrient changes will regulate the C, N, and P stoichiometry of plants. Wildfire changes the concentrations of soil nutrients, which usually occur during the early recovery periods (Chen et al., 2015). Hence, we hypothesized that the plant C, N, and P stoichiometry allocations would be substantially changed in the early periods during recovery (H_1_, Figure 1).

The responses of C, N, and P stoichiometries in plants to soil nutrient alterations will affect species composition (Li et al., 2012; Zhang et al., 2012); therefore, ultimately elemental allocations among plant organs are observed not only at the species level but also at the community level (Enquist, 2002; Elser et al., 2010). Previous studies have reported that nutrient allocation among plant organs is not constant and is regulated by soil and litter nutrients (Yan et al., 2016; Zhang Q. et al., 2018). Hence, to fully understand the effects of the nutrient changes on plant physiology after a wildfire, it is necessary to reveal the allocation strategies between organs (Zhang et al., 2015). Allometry theory provides an approach to describe elemental distribution among plant organs at the species and community levels (Kerkhoff et al., 2006; Enquist et al., 2007). Based on the phylogeny of plants, the allometric scaling indicator was derived from a general model, i.e., *Y* = *bX^a^*, where *a* represents the sloping indicator (Enquist, 2002; Reich et al., 2010). This relationship has been used successfully in predicting the numerous physiological traits and nutrient utilizations from species to community levels (Gillooly and Allen, 2007; Zhang J. et al., 2018; Zhao et al., 2020). For example, according to the optimal allocation theory, plants growing in nutrient-rich environments will allocate more nutrients to the leaf to increase photosynthesis, while plants allocate more nutrients to the root to increase nutrient acquisition in infertile environments (Palmroth et al., 2013; Yang et al., 2015). The changes in restricted nutrients will also affect the C assimilation (Minden and Kleyer, 2014; Freschet et al., 2015), which further influences species diversity and vegetation community (Sterner and Elser, 2002). Therefore, due to the alterations of nutrients in the burned area, we hypothesized that the C, N, and P allocations between leaf and fine root will change during the different recovery periods at the species and community level (H_2_, Figure 1).

The change in C, N, and P allocation strategies with the ecosystem recovery is vital but unclear. Boreal forests are an important part of the global total carbon pool (Shuman et al., 2011). Frequent occurrences of wildfires in boreal forests had profound effects on plant nutrient utilization, ecosystem structure, and functioning in the forest ecosystems (Wu et al., 2013). Thus, understanding the nutrient circulation of fire-prone boreal forests is a key issue for postfire management (Liu et al., 2012a; Wu et al., 2014). As an important part of boreal forests, the Great Xing’an Mountains of northeastern China host the southern extension of the larch forests and account for 30% of China’s timber production (Wang et al., 2010). Hence, our study aims to address the abovementioned knowledge gap of plant nutrient adaptation under moderate fire severity in boreal forests in northeast China. The study sought to answer the following scientific questions: (1) How do the C, N, and P stoichiometries of leaf and fine root change during recovery periods? (2) How do the plant C, N, and P allocations between leaf and fine root change during the recovery period? (3) What is the difference in elemental allocation at the species and community levels during the ecosystem recovery?

## Materials and Methods

### Site Description

This study was conducted in the Xilinji Forestry Bureau, which belongs to Mohe city in the Great Xing’an Mountain area of northeastern China. The climate is characterized by a long and severe winter, with mean annual air temperature ranging from –6°C to 1°C (Hu et al., 2017), and the mean annual precipitation is 500 mm. The dominant tree species are the *Larix gmelinii (Rupr.)* Kuzen, *Pinus sylvestris* Linn. var. *mongholica* Litv., *Picea koraiensis* Nakai, *Betula platyphylla* Suk., and two species of aspen (*Populus davidiana* Dode and *Populus suaveolens* Fisch.). Understory shrubs are dominated by *Ledum palustre* Linn., *Vaccinium vitis-idaea* Linn., *Rhododendron dauricum* Linn., *Vaccinium uliginosum* Linn., and *Eriophorum angustifolium* Honck (Meng et al., 2017). Soils are classified as brown soil (Wrb Iwg, 2015). The Great Xing’an Mountains are usually affected by natural wildfire disturbances due to the accumulation of combustible matter on the forest floor. Fire regimes are characterized by surface fires mixed with stand-replacing crown fires (Liu et al., 2012a). Records of fire provided us with the background to undertake experiments in this area.

### Experimental Design

Fire severity refers to the severity of organic material consumed or vegetation mortality directly caused by fire (Lentile et al., 2006). Fire severity follows the standard of Composite Burn Index (CBI) assessment protocol (Key, 2006; Lentile et al., 2006). Specifically, we visually estimated the changes in coarse woody debris, black carbon, char height, mortality rates of tall trees, and the proportions of fallen trees (Key, 2006; Boby et al., 2010; Fang and Yang, 2014). After the investigations, we found that the burn proportion was 41–60% and was viewed as moderate fire severity. Based on the precise historical records, we selected five treatments with different recovery periods, including four burned treatments (the fire occurred in 2015, 2007, 1997, and 1987, respectively) and one unburn treatment, and each treatment had three replications (three plots per treatment). The interval of each plot was more than 100 m to avoid the spatial autocorrelation between plots. During July and August 2017, 15 plots (20 × 20 m) were established, with 12 plots in the burned treatments (2a, 10a, 20a, and 30a, respectively) and the other 3 plots in the unburned treatment. The treatments were presented as at year 2, year 10, year 20, and year 30 after recovery and unburned treatment hereafter. In our study, 2a and 10a were viewed as early recovery periods, 20a and 30a as the medium recovery periods, and unburned treatment as the long recovery period. Considering the effects of environmental characteristics, there were no significant differences in soil bulk, slope aspect, slope position, and altitude among the selected plots (more details of the data information in Supplementary Table 1). The study area is located in the cold temperate continental climate. The forest type of treatment was *L. gmelinii* forest. These treatments had similar topography and environmental factors.

### Field Sampling

#### Plant Sampling

We investigated the species in each plot, obtained data on diameter at breast height (DBH), height, multiplicity of all trees, canopy density, and multiplicity of shrubs and herbs to calculate species diversity and the importance value of each species. We sampled all species in each quadrat (Species information in Supplementary Table 1), in which three replicate individuals of every healthy species were selected. We obtained 30 plant samples from the unburned treatment, 39 plant samples from 2a, 48 plant samples from 10a, 36 plant samples from 20a, and 30 plant samples from 30a. Plant samples were collected in August 2017. The fresh intact current-year leaves of each individual species were sampled from four orientations (the north and south, east and west) at the middle and low height of the tree canopy, and then the leaves were mixed into one leaf sample (*ca.* 80–100 g). We manually dug a hole within the depth of 2 m and grubbed the fine roots (diameter < 2 mm, *ca.* 40–60 g) from each species. The leaf and fine root samples of each species were obtained from the same individual. The samples were kept at 4°C and quickly transported to the laboratory. Plant samples (including leaf and fine root) were cleaned carefully with distilled water and then oven-dried at 65°C to constant weight in the laboratory. The samples were ground to 0.15 mm for chemical analyses.

### Soil and Litter Sampling

Five litter samples were collected from five points (four vertices and the center) in each plot. The aboveground plant material and live roots were removed before soil sampling. Three soil cores were collected from each point at a depth of 0–20 cm using a metal auger with an inner diameter of 5 cm. Fresh samples were placed in polyethylene ziplock bags, stored in a cooler with ice, and transported to the laboratory within 8 h, where they were preserved at 4°C. The chemical analyses of the soil and litter samples were completed in 10 days after sampling.

### Samples Analyses

#### Chemical Analyses

The total C and N concentrations of the plant (leaf and fine root), litter, and soil were measured with an elemental analyzer (Vario MAX CN Elemental Analyzer, Elementar, Hanau, Germany). The total P concentrations of plant, litter, and soil were measured using the ammonium molybdate method with a continuous-flow analyzer (AutoAnalyzer 3, Bran Luebbe, Hamburg, Germany), after Se-CuSO_4_-K_2_SO_4_-H_2_SO_4_, H_2_SO_4_-H_2_O_2_, and H_2_SO_4_-H_2_O_2_ digestion for soil, litter, and plant samples, respectively.

#### Data Analyses

Reduced major axis (RMA, also called standardized major axis) regression was used to determine the sloping indicator and constant of the log–log-linear functions (Warton et al., 2010). The data of C concentration in leaf and fine root were log-transformed. The allocation relationship of C in leaf and fine root was described by the equation as follows:


$$\text{Log}\,\left(Y\right)=\text{log}\;\,\left(a\right)+b^{*}\,\text{log}\;\,\left(X\right),$$

where *X* is the total C concentration of leaf, *Y* is the total C concentration of fine root, *a* is the intercept on the y-axis, and *b* is the slope of the linear equation, which represents the allometry exponent (Supplementary Tables 2, 3). When *b* = 1, the relationship of *X* to *Y* is isometric; otherwise, the relationship is allometric. If *b* > 1, *Y* changes more than *X*, whereas *b* < 1 indicates that *X* changes more than *Y* (Warton and Weber, 2015).

The sloping relationships of the elemental concentrations between fine root and leaf were analyzed at species and community levels. At the species level, we explored the sloping relationship of the elemental concentration using the log-transformed elemental concentrations of the fine roots and leaves of all species. The significant level for testing slope heterogeneity and differences from slope = 1 was *P* < 0.05. Differences in the regression slopes among different recovery periods were tested using multiple *post-hoc* comparisons (Duncan’s tests). Similar analyses were conducted for N and P between leaf and fine root at different recovery periods.

The Importance Value Index (IV) is calculated based on the relative dominance (Dr) through the basal area, the relative frequency (Fr) by the presence of the species, relative height (Hr) through the tree height, and relative coverage (Cr) according to the number of trees per unit area (Zhao et al., 2020):


Tree’⁢s⁢IV=(Hr+Fr+Dr)/3;

⁢Shrub’⁢s⁢IV=(Dr+Cr)/2;

⁢Herb’⁢s⁢IV=(Dr+Cr)/2

At the community level, the elemental concentrations of leaf and fine root were calculated using importance value (*IV*) weighted averages as follows:


$$E\,\text{com}=\sum_{i\,1}^{n}\left(E\,i\times I\,V\,i\right)/\sum_{i=1}^{n}I\,V\,i,$$

where *Ei* (g/kg) is the elemental concentration of the *i*th species in a quadrant and *IVi* is the importance value of the *i*th plant species. The investigated data were used for *IV* measurement. *E*com (g/kg) is the elemental concentration at the community level. The analyses of scaling relationships of elemental concentrations at the community level were the same as those at the species level. All statistical analyses were performed using the package of “*smatr*” in R 3.3.2.

Differences in elemental concentrations in the litter and soil in different recovery periods were tested using analysis of variance (ANOVA) with multiple comparisons of Duncan’s *post-hoc* tests using the general linear regression model. The significant level was set at *P* < 0.05. All statistical analyses were performed using R 3.3.2 statistical software (R Core Team, 2017). All graphs were generated by SigmaPlot (Systat Software, San Jose, CA, United States, 2017).

## Results

### Changes of Soil and Litter Nutrients in Recovery Periods

**FIGURE 2:**
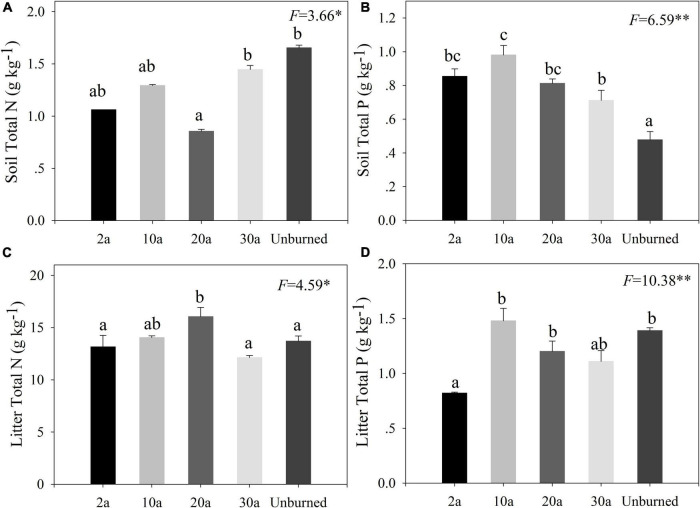
Changes of soil and litter N and P concentrations in different recovery periods. Error bars are standard errors. Different lowercase letters indicate significant differences among different recovery periods (**P < 0.01; *P < 0.05). 2a, 10a, 20a, and 30a are at year 2, year 10, year 20, and year 30 after recovery, respectively. The number of the data involved in analysis of variance (ANOVA) in each recovery period was as follows: soil *n* = 9 and litter: *n* = 15. **(A,B)** The pattern of soil. **(C,D)** The pattern of litter.

Compared with the unburned treatment, the soil N concentration decreased after a wildfire (Figure 2A, *P* < 0.05), whereas soil P concentration significantly increased (Figure 2B, *P* < 0.05), especially in early periods of recovery (2a and 10a). The litter N concentration decreased at 2a and was the highest at 20a recovery (Figure 2C, *P* < 0.05). The litter P concentration significantly decreased at 2a compared with the unburned area and then increased at 10a (Figure 2D, *P* < 0.05).

### Changes in Leaf and Fine Root Stoichiometry of C, N, and P in Recovery Periods at the Species Level and the Community Level

**FIGURE 3:**
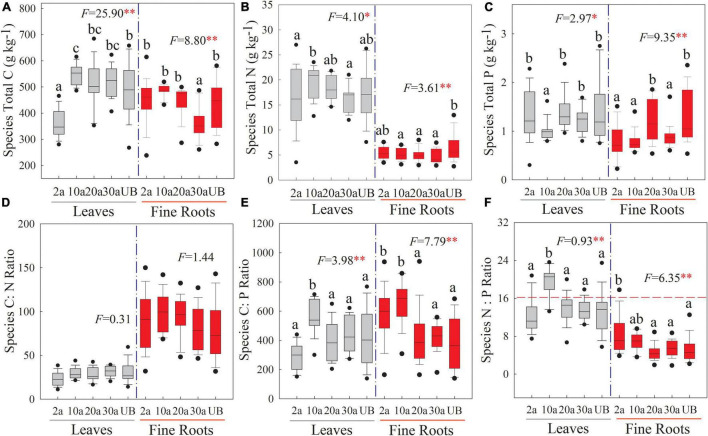
Changes in the stoichiometry of leaf and fine root at the species level in different recovery periods. Boxplots stand for the range of C, N, and P stoichiometry during recovery periods. The dashed horizontal line stands for N:P ratio = 16 above which indicates the P limitation in subfigure (f). 2a, 10a, 20a, and 30a are at year 2, year 10, year 20, and year 30 after recovery, respectively. UB, unburned. Error bars are standard errors. Different lowercase letters indicate significant differences among different recovery periods. The number of the data involved in ANOVA in each recovery period was as follows: 2a: *n* = 39, 10a: *n* = 48, 20a: *n* = 36, 30a: *n* = 30, UB: *n* = 30 (**P < 0.01; *P < 0.05). **(A–C)** The stoichiometry concentration. **(D–F)** The stoichiometry ratio.

At the species level, the C, N, and P stoichiometries showed significant differences among five recovery periods (Figure 3, *P* < 0.05), and the concentrations of these elements were higher in the leaf than in the fine root. The leaf C concentration was significantly lower at the early recovery period (2a) than those at other recovery periods, whereas fine root C concentration was significantly lower at the medium recovery period (30a; Figure 3A, *P* < 0.01). Leaf N was higher at 10a recovery. Compared with unburned, fine root N concentration significantly decreased after a wildfire (Figure 3B, *P* < 0.05). The P concentration of leaf significantly decreased at 10a (Figure 3C, *P* < 0.05). The C:P ratio of leaf and fine root significantly increased at 10a (Figure 3E, *P* < 0.01). Moreover, the N:P ratio of the leaf significantly increased at 10a. The mean value of leaf N:P ratios was lower than 14 under the unburned treatment but was higher than 16 at 10a (Figure 3F, *P* < 0.01).

**FIGURE 4:**
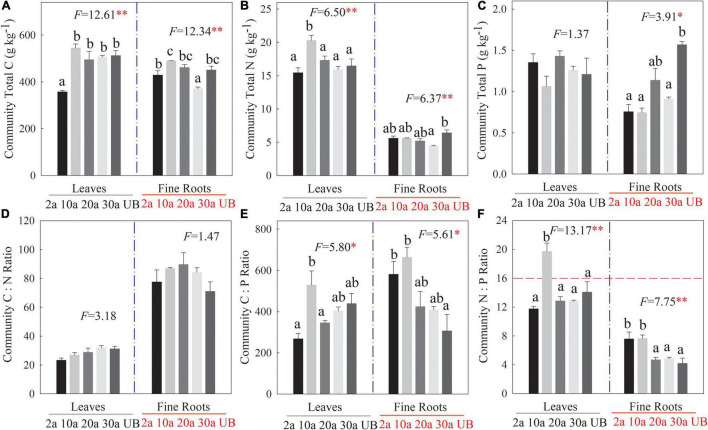
Changes in C, N, and P stoichiometry of leaf and fine root at community level among different recovery periods. 2a, 10a, 20a, and 30a are at year 2, year 10, year 20, and year 30 after recovery, respectively. UB, unburned. Error bars are standard errors. Different lowercase letters indicate significant differences among different recovery periods. The dashed horizontal line stands for N:P ratio = 16 above which indicates the P limitation in subfigure (f). The number of the data involved in ANOVA in each recovery period was as follows: 2a: *n* = 9, 10a: *n* = 9, 20a: *n* = 9, 30a: *n* = 9, UB: *n* = 9 (**P < 0.01; *P < 0.05). **(A–C)** The stoichiometry concentration. **(D–F)** The stoichiometry ratio.

The C, N, and P stoichiometries at the community level showed variations among five recovery periods (Figure 4). Compared with the unburned treatment, leaf C concentration significantly declined at 2a, while the C concentration of fine root decreased significantly at 30a (Figure 4A, *P* < 0.01). Similarly, the leaf N concentration at the community level was the highest at 10a. However, fine root N concentration decreased significantly at 30a as compared with unburned treatment (Figure 4B, *P* < 0.05). The fine root P concentration decreased significantly at 10a compared with the unburned treatment (Figure 4C, *P* < 0.05). The leaf N:P ratios at the community level also increased significantly at 10a, and the mean value was higher than 16 (Figure 4F, *P* < 0.01).

### Sloping Relationships of C, N, and P Between Leaf and Fine Root in Recovery Periods at the Species Level and the Community Level

**FIGURE 5:**
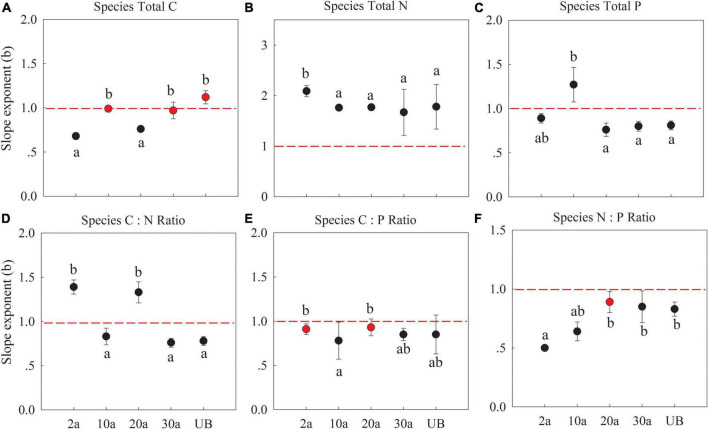
Slope exponents (b) of C, N, and P between fine root (Y) and leaf (X) at the species level. 2a, 10a, 20a, and 30a are at year 2, year 10, year 20, and year 30 after recovery, respectively. UB, unburned. The sloping exponents of five recovery periods were shown with standard error. The slope exponents were estimated using reduced major axis (RMA) regressions. The summary of RMA, including intercept (a) and *R*^2^, is shown in Supplementary Table 2. The number of the data involved in slope fitness in each recovery period was as follows: 2a: *n* = 39, 10a: *n* = 48, 20a: *n* = 36, 30a: *n* = 30, UB: *n* = 30. All regressions are significant at *P* < 0.05. Red dotted lines mean the slope is equal to 1. The red dots indicate that the exponents are not significantly different from 1 (isometric relationship) based on the likelihood tests. The different lowercase letters denote significant differences between the exponents within burned years based on the likelihood tests. **(A–C)** The stoichiometry concentration. **(D–F)** The stoichiometry ratio.

The C, N, and P allocation slope exponents of fine root vs. leaf at the species level were significantly different among the recovery periods (Figure 4 and Supplementary Table 1). The slopes of C concentration showed an allometry pattern (*b* < 1) at 2a after when it exhibited an isometry pattern (*b* = 1; Figure 5A, *P* < 0.05), indicating that the allocation of leaf C would change more in the early recovery periods. All slope exponents of N concentration were larger than 1 (*b* > 1), indicating that the allocation of root N would change more than the leaf (Figure 5B, *P* < 0.05). As for the P concentration, the slope exponents transformed from *b* > 1 in early recovery periods (10a) to *b* < 1 in medium recovery periods (Figure 5C, *P* < 0.01). The slope exponent of C:N in the long recovery period (unburned) decreased as compared with those of early recovery periods. The slope of C:P and N:P was generally smaller than 1.

**FIGURE 6:**
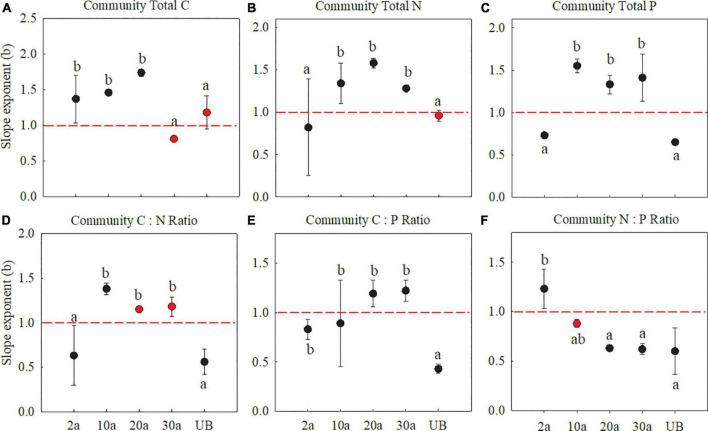
Slope exponents (b) of C, N, and P with fine root (Y) vs. leaf (X) at the community level. 2a, 10a, 20a, and 30a are at year 2, year 10, year 20, and year 30 after recovery, respectively. UB, unburned. The sloping exponents of five recovery periods were shown with standard error. The slope exponents were estimated using RMA regressions. The summary of RMA, including intercept (a) and *R*^2^, is shown in Supplementary Table 3. The number of the data involved in slope fitness in each recovery period was as follows: 2a: *n* = 9, 10a: *n* = 9, 20a: *n* = 9, 30a: *n* = 9, UB: *n* = 9. All regressions are significant at *P* < 0.05. Red dotted lines stand for the slopes are equal to 1. The red dots are that the exponents are not significantly different from 1 (isometric relationship) based on the likelihood tests. The different lowercase letters denote significant differences based on the likelihood tests. **(A–C)** The stoichiometry concentration. **(D–F)** The stoichiometry ratio.

At the community level, C, N, and P allocation slope exponents between fine root and leaf also showed differences among the five recovery periods. The allocation slope exponents of C concentration between fine root and leaf showed allometry (*b* > 1) after burn (Figure 6A, *P* < 0.05). As for N and P concentrations, the slope exponents (*b*) were smaller than 1 at 2a then transformed to larger than 1 after 10a recovery (Figures 6B,C, *P* < 0.05). Interestingly, all slope exponents of plant C, N, and P concentrations at medium-term recovery (20a) showed clear allometry (*b* > 1), which further exhibited a transformation from *b* < 1 to *b* > 1 at 20a and 30a. This transformation indicated that more C, N, and P changes in fine root with an increased recovery period in comparison with those in leaf at the community level (Figures 6A–C, *P* < 0.05). The slopes of C:N and C:P all showed an allometric pattern, which transformed the slope from smaller than 1 to larger than 1 with increasing recovery periods.

## Discussion

The results demonstrated that wildfires altered the soil and litter nutrients and consequently changed plant C, N, and P stoichiometries during the recovery periods. Plant allocated more nutrients to fine roots than leaves at the species level, whereas more nutrients were allocated to the leaves at the community level in early recovery periods (i.e., 2a and 10a). Subsequently, the plant allocated more N and P to fine roots with increasing recovery periods at the community level, which highlights the effect of wildfire on the elemental allocation strategies that differ between species level and community level.

### Changes of Soil and Litter Nutrients Among Recovery Periods

In our study, the changes in soil and litter nutrient concentrations largely varied among the different recovery periods, for example, litter N, litter P, and soil N concentrations decreased at year 2 after recovery. Wildfire altered the soil nutrient pools and reduced litter nutrient concentrations (Tufekcioglu et al., 2010; Michalzik and Martin, 2013; Holden et al., 2016). Wildfire incinerates the majority of litter on the forest floor, where the amount of nutrients volatilizes with the destroyed vegetation (Toberman et al., 2014; Scalenghe et al., 2015). Limited nutrient supplement hampers aboveground growth and lowers the nutrient concentrations of litter (Nobles et al., 2009). Soil total N decreased in early recovery periods, which is consistent with a previous study that wildfires with moderate severity caused the declines in soil N pool (Wan et al., 2001). In contrast, significant increases in soil P concentration were observed in our study, which may be because the P is hard to volatilize under moderately severe fires, and fires can promote the P released from soil (Butler et al., 2018). The soil and litter N concentrations were increased in the medium recovery periods (Figure 2). With increasing recovery periods, the increases of soil and litter N may be caused by the restoration of the understory community, which is consistent with the increasing soil and litter nutrient concentrations with higher species diversity (Scalenghe et al., 2015).

### Differential Stoichiometries of C, N, and P Among Recovery Periods

An important issue of plant growth is its response to the changes of N and P supplements in burned ecosystems (Tarvainen et al., 2016). The total C, N, and P stoichiometries of leaf and fine root showed variations among five recovery periods regardless of the levels (Figures 3 and 4). The most striking result was that the stoichiometry of the leaf and fine root at year 10 after recovery was significantly different from others. At the species and community levels, leaf N concentrations were the highest, and the P concentrations of leaf and fine root were the lowest at year 10 after recovery. Although soil nutrients substantially decreased after being burned, plants adopt a growth strategy in response to severe environmental changes (Song et al., 2021). These flexible growth strategies of the plant would change resource allocations among organs to enable them to adapt to the nutrient-insufficient environment (Wright and Sutton-Grier, 2012; Song and Liu, 2019). The results of our study show that the C, N, and P concentrations were higher in leaf than in fine roots. Plants usually promote the photosynthetic efficiency of the leaf and allocate more resources to the aboveground (Ordoñez et al., 2009), which promotes leaf N utilization in our study. Additionally, to fully use light after a wildfire, plants can promote leaf photosynthesis and allow more nutrient investments to leaf (Muqaddas et al., 2015), which can be demonstrated in the higher litter nutrient concentration in our results. Leaf N absorption efficiency rapidly increases during the period of nutrient insufficiency, and thus, plants significantly promote leaf N concentrations (Scoffoni et al., 2011).

The decline of plant P concentration at year 10 after recovery may be due to the massive appearance of regenerating species that have low P concentrations (Yu et al., 2011; Shenoy et al., 2013). Moreover, in early recovery periods, soil organic acid secretion increases soil acidification, causing a huge loss in soil available P (Butler et al., 2017), which then decreases root P concentrations (Hume et al., 2016). Compared with unburned treatment, leaf C concentration was significantly decreased at year 2 after recovery, whereas fine root C concentration increased (Figures 3 and 4). At the species and community levels, a previous study also observed that the aboveground biomass decreased during early recovery periods (Nave et al., 2011). In the context of soil nutrient deficiencies, the regenerating tree species will synthesize more C to root growth to absorb nutrients under disturbance conditions (Mo et al., 2010; Kong et al., 2015).

The plant C:nutrient ratios are not constant after wildfires (Cui et al., 2010; Pellegrini et al., 2015), which was also observed in our results with significant changes of C:P and N:P ratio, especially at year 10 after recovery. More importantly, the N:P ratio at the species and community levels showed a similar pattern that the leaf N:P ratio was higher than 16 at year 10 after recovery, suggesting that plant growth tended to be a P limitation. This result of leaf N:P ratios was consistent with the previous observation under fire (Bai et al., 2013). N and P are usually the limiting nutrients for plant growth in forest ecosystems (Bünemann et al., 2018). According to the eco-stoichiometry theory, the higher leaf N:P ratio leads to a lower concentration of mRNA, thereby suggesting that species growth may likely be limited by P deficiency (Güsewell, 2004). During the recovery period, the appearance of regenerating species enhances the leaf N concentration, but leaf P concentration tends to be lower (Certini, 2005). After a decade’s recovery from wildfire, the recovery of N and P suggests that community restoration attenuates P limitation in plant growth (Wood and Bowman, 2012; Dantas Vde et al., 2016). Thus, the nutrient changes in different recovery periods may contribute to the allocation strategies of plant C, N, and P.

### Differential Allocation of C, N, and P Between Leaf and Fine Root Between Species Level and Community Level in Early Recovery Periods

As mentioned above, plants can adjust the elemental concentrations according to the nutrient supply, especially in early recovery periods. These stoichiometry shifts would further affect the nutrients’ reallocation between above- and belowground parts (Niklas, 2005; Schafer and Mack, 2010). Allocation slope exponents of fine root vs. leaf at the species and community levels were significantly different among recovery periods (Figures 5 and 6). At the species level, the allocation slopes of N and P concentrations were significantly larger than 1 during the recovery period of 2 years and 10 years, indicating that more nutrient changes occur in the fine root than a leaf in early recovery periods. The changes in the element distribution of leaf and fine root largely drive the pattern of organs’ metabolic activity and their functions (Kerkhoff et al., 2006). Leaf functioning usually depends on the nutrients offered by the roots, and at the same time, root growth relies on the carbohydrates produced by the leaf (Minden and Kleyer, 2014; He et al., 2016). Under low soil N concentrations, more nutrients will be allocated to the root to sustain vital physiological functions to mine nutrients (Fortunel et al., 2012; Yan et al., 2016). Another possible reason is that the leaf needs more nutrients to ensure photosynthesis, which requires higher nutrient investments to roots to mine nutrients (Marschnert et al., 1997). Increased soil P concentrations could promote root growth. Thus, at the species level, nutrients were more changed in fine root than a leaf in early recovery periods.

At the community level, the slope of C, N, and P allocation between leaf and fine roots was smaller than 1 at year 2 after recovery, indicating that more elements changed in leaf than fine root (Figure 6). Species diversity and community composition are being strongly influenced by the nutrient changes (Liu et al., 2012b), especially after a wildfire (Nave et al., 2011). In the early recovery periods, greater species diversity with the occurrence of regeneration species presents fast growth strategies and high nutrient utilization. Even under low soil nitrogen concentrations, more N and P were allocated to aboveground at the community level (Maliakal et al., 2000; Niklas, 2005). Further evidence shows that the increasing soil P concentration after wildfire promotes more nutrient changes in the leaf of regenerated species, such as herbs with a shorter leaf life span (Johnstone et al., 2010; Chen et al., 2013). The regenerated plants need to enhance their photosynthetic rate to obtain light during their short growing season (Elser et al., 2010). Compared to woody species, herbaceous species were characterized by more leaf nutrient changes and quick growth, which can boost community recovery (Adler et al., 2014).

### Transformed Nutrient Allocation at the Community Level With an Increasing Recovery Period

At the community level, the N and P allometry slope between leaf and fine root showed a clear transformation with the increasing recovery periods. Specifically, all slope exponents of N and P concentration showed allometry (*b* < 1) in early recovery periods (i.e., 2a and 10a), after when the slope exponents were more than 1 at medium recovery period (Figure 6). This transformation of slope exponents indicated that more N and P would change in fine root in the medium recovery periods (i.e., 20a and 30a). At the community level, the nutrient allocation in early recovery periods showed more changes of nutrient in leaf than fine root, which is similar to the finding that shrubs would preferentially allocate P to leaf to maintain plant physiological functions (Fortunel et al., 2012). From early- to medium-term recovery, the species diversity would enrich with the community recovery. The nutrient allocation relationship would be changed at the community level (Chen et al., 2019). The dynamics of nutrients at the community level reflect the combined results of nutrient allocation in the ecosystem (Lavorel and Grigulis, 2012). In medium recovery periods (i.e., 20a and 30a), soil N and P concentrations were recovered to those of unburned treatment; therefore, more nutrients will be transported to nonphotosynthetic organs to promote plant growth and enhance plant competitiveness (Fortunel et al., 2012), especially in the regenerated shrub species (Lavorel, 2013). Plants may allocate more nutrients to the stems and roots than the leaf to survive in nutrient-limited conditions (Enquist, 2002). Even in areas where soil N content is relatively abundant, more N is transported to roots to promote community stability (Fortunel et al., 2012). Thus, more nutrients are allocated to the leaf in early recovery periods, and more nutrients are allocated to root in medium recovery periods, which is consistent with our hypothesis (H_2_).

Nutrient allocation is important for plants to adapt to environmental changes, which may be of particular importance for plant resource distribution in response to nutrient changes (Ordoñez et al., 2009; Smithwick et al., 2012). Therefore, the findings in our study suggest that differentially elemental allocation of a plant is crucial for resource utilization during ecosystem recovery after the wildfire.

## Conclusion

In our study, we demonstrated that the N and P concentrations at year 10 after recovery were significantly different from other times at the species and community levels due to the changes in soil and litter nutrients, especially in early recovery periods. Specifically, at the species level, more changes of nutrients occur in the fine root than in the leaf, while more changes of nutrients occurred in the leaf at the community level. Additionally, more changes in the nutrients were observed in fine roots during the medium recovery periods at the community level. Plant growth tends to P limitation at 10 years of recovery. This study emphasized the importance of the C, N, and P allocation strategies of leaf and fine root and differed among recovery periods at the species and community levels, which will promote the understanding of plant adaptation during forest ecosystem restoration.

## Data Availability Statement

The raw data supporting the conclusions of this article will be made available by the authors, without undue reservation.

## Author Contributions

ZS carried out the analyses and wrote the manuscript. YLi designed this research. ZL, YLu, and XW revised the manuscript. All authors were heavily involved in writing.

## Conflict of Interest

The authors declare that the research was conducted in the absence of any commercial or financial relationships that could be construed as a potential conflict of interest.

## Publisher’s Note

All claims expressed in this article are solely those of the authors and do not necessarily represent those of their affiliated organizations, or those of the publisher, the editors and the reviewers. Any product that may be evaluated in this article, or claim that may be made by its manufacturer, is not guaranteed or endorsed by the publisher.
