# Supplementary material for: Allocation Strategies of Carbon, Nitrogen, and Phosphorus at Species and Community Levels With Recovery After Wildfire
Source: Front Plant Sci. 2022 Apr 11;13:850353. doi: 10.3389/fpls.2022.850353 (PMC9037545; doi:10.3389/fpls.2022.850353)
Supplement: Supplementary file 1 [file Table_1.DOCX]

**SUPPLEMENTARY TABLES**

**TABLE S1** | The background of experimental sites and soil condition

| Burn  Recovery | Site Coordinates | Soil bulk /  g cm^-3^ | Slope aspect  (position)/  ° | Altitude/  m | Species number | Species |
| --- | --- | --- | --- | --- | --- | --- |
| 2a | 52°32′01″ N 122°38′15″ E | 0.75A | 12A (SE) | 426A | 10 | *Larix gmelinii (Ruprecht)* Kuzeneva; *Betula platyphylla* Sukaczev; *Ledum palustre* Linn; *Vaccinium uliginosum* Linn.; *Rosa acicularis* Lindl.; *Vaccinium uliginosum* Linn.; *Ribes nigrum* Linn.; *Buxus sinica var. pumila* M. Cheng.; *Quercus wutaishanica* Blume,; *Lathyrus palustris* Linn. |
| 10a | 52°32′11″ N  122°38′19″ E | 0.76A | 9A (E) | 440A | 13 | *Ledum palustre* Linn*; Betula fruticosa* Pall.*; Vaccinium uliginosum* Linn.*; Larix gmelinii (Ruprecht)* Kuzeneva*; Betula platyphylla* Sukaczev*; Rosa acicularis* Lindl.*; Vaccinium uliginosum* Linn.*; Alnus mandshurica* Mazz. *; Ribes nigrum* Linn.*; Filipendula palmata* (Pall.) Maxim. et al |
| 20a | 52°33′39″ N  121°45′46″ E | 0.74A | 11A (SE) | 428A | 16 | *Sambucus williamsii* Hance*; Deyeuxia langsdorffii* (Link) Kunth.; *Ledum palustre* Linn; *Vaccinium uliginosum* Linn.; *Larix gmelinii (Ruprecht)* Kuzeneva; *Betula fruticosa* Pall.; *Rhododendron dauricum* Linn.; *Betula platyphylla* Sukaczev; *Vaccinium uliginosum* Linn.; *Rosa acicularis* Lindl.; *Deyeuxia langsdorffii* (Link) Kunth; et al |
| 30a | 52°54′39″ N  122°30′29″ E | 0.71A | 10A (SE) | 430A | 12 | *Vaccinium uliginosum* Linn.; *Ledum palustre* Linn; *Larix gmelinii (*Ruprecht) Kuzeneva; *Betula fruticosa* Pall.; *Betula platyphylla* Sukaczev; *Vaccinium uliginosum* Linn.; *Rosa acicularis* Lindl.; *Deyeuxia langsdorffii* (Link) Kunth; *Filipendula palmata* (Pall.) Maxim. et al |
| Unburned | 53°28′31″ N  122°18′51″ E | 0.68A | 9A (E) | 450A | 10 | *Larix gmelinii (Ruprecht)* Kuzeneva*; Ledum palustre* Linn*; Pyrola dahurica (H. Andr.)* Kom*.; Populus davidiana* Dode*; Betula fruticosa* Pall.*; Betula platyphylla* Sukaczev*; Pinus sylvestris* Linn.*; Salix myrtilloides* Linn.*; Vaccinium uliginosum* Linn. et al |
|  |  | *F*=0.61^ns^ | *F*=0.91^ns^ | *F*=0.67^ns^ |  |  |

2a, 10a, 20a, 30a are at year 2, year 10, year 20, and year 30 after recovery, respectively. The capital letter “A” indicates no significate different between recovery periods, *P*<0.05. SE represents the slope of south east, E represents the slope of east.
