# Supplementary material for: Allocation Strategies of Carbon, Nitrogen, and Phosphorus at Species and Community Levels With Recovery After Wildfire
Source: Front Plant Sci. 2022 Apr 11;13:850353. doi: 10.3389/fpls.2022.850353 (PMC9037545; doi:10.3389/fpls.2022.850353)
Supplement: Supplementary file 2 [file Table_2.docx]

**TABLE S2** | Reduced major axis (RMA: Y=ax^b) regression results of C, N, and P concentrations and their ratio between leaf and fine root at species level

| Fine root vs. Leaf  (Y vs. X) | Recovery  periods | b | 95%CI | a | 95%CI | R^2^ | *P* |
| --- | --- | --- | --- | --- | --- | --- | --- |
| Total C | 2 | 0.68a | (0.48,0.96) | -0.75 | (-1.39,-0.12) | 0.62 | 0.03 |
|  | 10 | **0.99b** | (0.15,1.82) | -1.62 | (-2.66,-0.42) | 0.61 | 0.01 |
|  | 20 | 0.76a | (0.44,1.04) | -1.42 | (-2.30,-0.54) | 0.54 | 0.002 |
|  | 30 | **0.97b** | (0.45,1.68) | -1.01 | (-1.68,-0.32) | 0.42 | 0.04 |
|  | UB | **1.12b** | (0.76,1.63) | -0.26 | (-1.39,0.87) | 0.55 | 0.02 |
| Total N | 2 | 2.09b | (1.49,2.91) | -0.34 | (-0.85,-0.18) | 0.58 | 0.000 |
|  | 10 | 1.76a | (1.49,2.02) | -0.67 | (-1.11,-0.39) | 0.42 | 0.02 |
|  | 20 | 1.77a | (1.59,1.94) | -0.74 | (-1.03,-0.54) | 0.50 | 0.07 |
|  | 30 | 1.67a | (0.46,1.97) | -0.77 | (-0.94,-0.59) | 0.42 | 0.03 |
|  | UB | 1.78a | (0.53,2.15) | -0.61 | (-0.86,-0.36) | 0.39 | 0.02 |
| Total P | 2 | 0.89ab | (0.63,1.26) | -0.03 | (-0.14,0.07) | 0.49 | 0.01 |
|  | 10 | 1.27b | (0.74,2.19) | -0.19 | (-0.36,0.09) | 0.80 | 0.004 |
|  | 20 | 0.76a | (0.49,1.18) | -0.17 | (-0.23,-0.10) | 0.36 | 0.04 |
|  | 30 | 0.80a | (0.54,1.17) | 0.05 | (-0.001,0.1) | 0.33 | 0.03 |
|  | UB | 0.81a | (0.54,1.18) | -0.04 | (0.13,-0.05) | 0.56 | 0.04 |
| C : N Ratio | 2 | 1.39b | (1.00,1.94) | -1.29 | (-2.21,-0.37) | 0.89 | 0.04 |
|  | 10 | 0.83a | (0.49,1.36) | -0.09 | (-0.95,0.23) | 0.72 | 0.04 |
|  | 20 | 1.33b | (0.88,2.02) | -0.08 | (2.96,5.21) | 0.59 | 0.05 |
|  | 30 | 0.76a | (0.52,1.10) | 0.06 | (-0.49,0.62) | 0.87 | 0.01 |
|  | UB | 0.78a | (0.53,1.13) | 0.02 | (-0.55,0.58) | 0.59 | 0.02 |
| C: P Ratio | 2 | **0.91b** | (0.65,1.29) | -0.07 | (-0.98,0.83) | 0.71 | 0.004 |
|  | 10 | 0.78a | (0.59,0.95) | -1.13 | (-3.34,1.08) | 0.81 | 0.004 |
|  | 20 | **0.93b** | (0.60,1.45) | -0.50 | (-0.88,-0.13) | 0.74 | 0.03 |
|  | 30 | 0.85ab | (0.58,1.26) | -0.84 | (-0.95,-0.73) | 0.79 | 0.002 |
|  | UB | 0.85ab | (0.68,1.46) | -0.39 | (-0.47,-0.25) | 0.73 | 0.03 |
| N:P Ratio | 2 | 0.50a | (0.35,0.70) | -1.50 | (-1.66,-1.33) | 0.65 | 0.001 |
|  | 10 | 0.64ab | (0.39,1.05) | -0.84 | (-1.55,-0.12) | 0.81 | 0.07 |
|  | 20 | **0.89b** | (0.58,1.38) | -0.69 | (-0.96,-0.42) | 0.74 | 0.02 |
|  | 30 | 0.85b | (0.30,1.67) | -0.82 | (-0.96,0.69) | 0.71 | 0.001 |
|  | UB | 0.83b | (0.56,1.22) | -0.54 | (-0.77,0.29) | 0.78 | 0.04 |

Regression slopes (b: Relationship of C, N, and P stoichiometry between leaf and root) in bold are not significantly different from 1. 2a, 10a, 20a, 30a are at year 2, year 10, year 20, and year 30 after recovery, respectively. UB is unburned. P value is for regression (all *P* < 0.05). Different letters indicate significant differences (*P* < 0.05) based on a likelihood ratio test. a_RMA_, regression intercept; CI, confidence interval.
